# Underlying factors determining grain morphologies in high-strength titanium alloys processed by additive manufacturing

**DOI:** 10.1038/s41467-023-38885-9

**Published:** 2023-06-06

**Authors:** Mohan S. K. K. Y. Nartu, Brian A. Welk, Srinivas A. Mantri, Nevin L. Taylor, Gopal B. Viswanathan, Narendra B. Dahotre, Rajarshi Banerjee, Hamish L. Fraser

**Affiliations:** 1 Center for Agile and Adaptive Additive Manufacturing, University of North Texas, Denton, TX 76207 USA; 2 Department of Materials Science and Engineering, University of North Texas, Denton, TX 76207 USA; 3 Center for the Accelerated Maturation of Materials, The Ohio State University, Columbus, OH 43210 USA; 4 Department of Materials Science and Engineering, The Ohio State University, Columbus, OH 43210 USA

**Keywords:** Metals and alloys, Engineering

## Abstract

In recent research, additions of solute to Ti and some Ti-based alloys have been employed to produce equiaxed microstructures when processing these materials using additive manufacturing. The present study develops a computational scheme for guiding the selection of such alloying additions, and the minimum amounts required, to effect the columnar to equiaxed microstructural transition. We put forward two physical mechanisms that may produce this transition; the first and more commonly discussed is based on growth restriction factors, and the second on the increased freezing range effected by the alloying addition coupled with the imposed rapid cooling rates associated with AM techniques. We show in the research described here, involving a number of model binary as well as complex multi-component Ti alloys, and the use of two different AM approaches, that the latter mechanism is more reliable regarding prediction of the grain morphology resulting from given solute additions.

## Abstract (web summary)

Predictive scheme for Ti alloys with equiaxed microstructures is often limited by the methods based on growth restriction factors, Q. Here, the authors present a predictive solution based on the freezing range of alloys for columnar to equiaxed transition during fusion-based additive manufacturing.

## Introduction

There has been much effort aimed at using additive manufacturing (AM) of titanium alloys such that attractive properties may be realized in net-shaped components^1–11^. The problem faced in most cases is that the microstructures of existing structural titanium alloys when processed using AM exhibit coarse columnar grains with fairly strong textures, which result in mechanical property deficits. There have been a number of approaches aimed at solving this problem associated with as-deposited AM microstructures of structural titanium alloys, including variations in processing parameters^8^, the use of inoculants^9,10^, alloying to effect a change in solidification mode, for example, attempting to exploit titanium alloys with peritectic reactions^11^, and dilute solute additions to existing titanium alloys to influence a columnar to equiaxed transition (CET) during solidification in AM processing^4–7,12^. While these various methodologies have exhibited some limited success regarding the AM processing of structural titanium alloys, it appears that the approach which has the most general application to AM of a number of titanium alloys involves the additions of dilute solute to effect a CET during processing.

The effective exploitation of this latter method of producing a CET during AM of titanium alloys would benefit greatly from the development of a predictive scheme for alloy selection and subsequent processing based on computational materials science and engineering. The latter requires accurate mechanistic details of the behavior of given alloys during processing and subsequent heat-treatment. Regarding the approach involving solute additions to existing titanium alloys, there appears to be some disagreement regarding the mechanism(s) at play during AM processing. Thus, in the published work on the Ti-Cu system^5^ and that involving TIMETAL Ti64 (Ti64: Ti-6Al-4V; *all compositions in this paper are in wt.%*) with dilute additions of Fe^6^, in both cases the choices of alloying additions were made on the basis of the values of the growth restriction factors, Q^13^. In this approach, a high value of Q would result in equiaxed microstructures whereas alloys with low values of Q would be expected to develop microstructures which would be columnar in nature, i.e., coarse with attendant relatively strong textures. The question that is not answered is at what value of Q does the CET occur, a value that would be essential for developing a predictive scheme.

In work which involved alloying CP-Ti with Fe, Ni, and Mo, the alloy Ti64 with additions of Fe and Ni, and the alloy Ti18 (Ti-5.5Al-5V-5Mo-2.4Cr-0.75Fe-0.15 O) with dilute additions of Fe^7^, the choice of alloying addition was made on the basis of the degree to which the freezing range of the given alloy would be increased by the given dilute solute addition. This approach is based on the notion that AM processing involves rapid rates of solidification. Hence, providing the alloy freezing range is sufficiently large, the rapid cooling rate imposed by AM would result in solidification initiating at a significant degree of undercooling. This in turn will result in a substantially high solid nucleation rate within the melt pool, and consequently a large number density of solid nuclei, which may result in a CET, leading to equiaxed grains^14^. This approach also suffers from the same problem noted above for the method based on growth restriction factors, namely that the actual degree of undercooling required to effect a CET is not known.

In this work, for dilute solute additions to Ti and Ti alloys processed by fusion-based additive manufacturing, we compare the accuracy of prediction of microstructure based on two competing mechanisms, either that based on growth restriction factors (Q) or that based on increasing the freezing range (ΔT). We show that regarding the prediction of effecting a CET by such dilute solute additions, the latter mechanism provides a more reliable predictive scheme for alloy development. Because in many cases calculations of both values of Q and ΔT are based on the use of computational thermodynamics (e.g., by use of the software packages Pandat™ and Thermocalc™), the quality of predictions will be subject to the degree of accuracy of the databases that underscore these predictive approaches. It is reasonable to expect that these databases will be most accurate for binary alloys, rather than more complex compositions, and so in the first part of the work described in the following, we have focused on binary alloys in an attempt to minimize one possibly significant source of uncertainty. Then, prediction of microstructures in more complex alloys processed by AM are considered. We have also used two fusion-based AM processes, one involving blown powder (directed energy deposition) and the other laser powder bed fusion to establish that the results obtained are due to the solute additions rather than factors associated with a given process. In the following, the same method of calculating the growth restriction factors in the Ti-Cu system^5^ is employed here such that the resultant factors are measures of *Q*_*bin*_^13^. In this way, direct comparisons may be made between the results previously presented for the Ti-Cu system^5^ and those described in this paper.

## Results and discussion

**Fig. 1:**
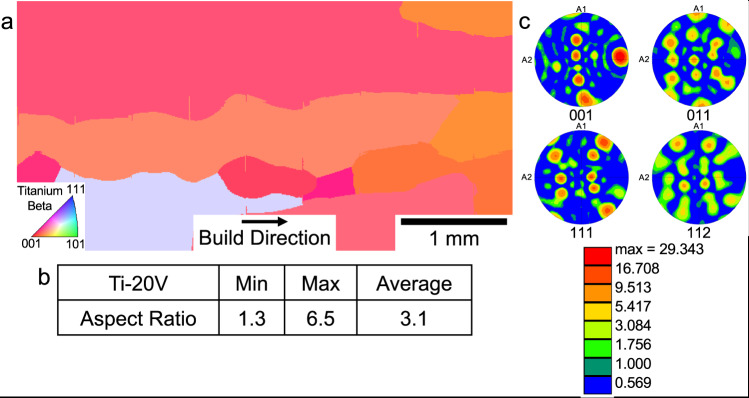
Characterization of the microstructure of the Ti-20V sample. **a** an IPF map obtained using EBSD to determine the coarse columnar grain morphology. **b** Results of image analysis to determine the average aspect ratios of the grains, yielding a result consistent with the columnar morphology. **c** Pole figures deduced from the EBSD experiment from which a moderate texture is observed.

**Fig. 2:**
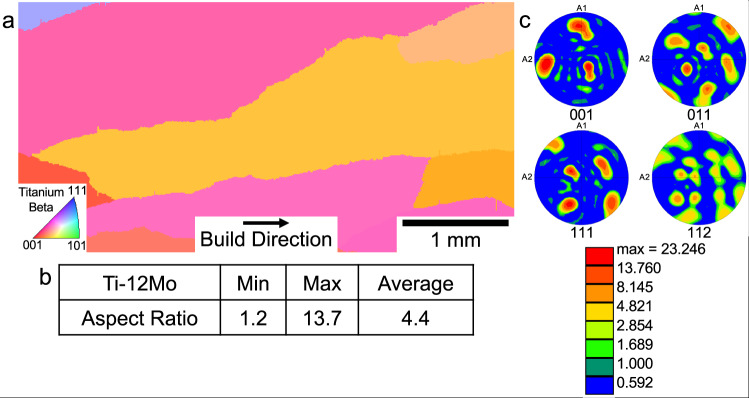
Characterization of the microstructure of the Ti-12Mo sample. a) an IPF map obtained using EBSD to determine the coarse columnar grain morphology. b) Results of image analysis to determine the average aspect ratios of the grains, yielding a result consistent with the columnar morphology. c) Pole figures deduced from the EBSD experiment from which a moderate texture is observed.

The first set of experiments in the present study was undertaken to determine the critical value of the growth restriction factor above which a CET would occur. Firstly, from the work presented for the alloy Ti-8.5Cu^5^, a value of 62 K was derived for the Q_bin_, which corresponded to an equiaxed grain morphology. In the present study, two alloys were chosen, one being Ti-20V, for which a value of Q was determined to be ≈0 K, i.e., very low, and the second Ti-12Mo, for which Q was estimated to be 54 K, quite close to the value of 62 K determined for Ti-8.5Cu^5^. Powders of both alloys were deposited in a Directed Energy Deposition (DED) system (specifically a LENS™ AM machine), and the resulting microstructures and textures of the as-built samples have been characterized. For the case of Ti-20V, the microstructure is shown in Fig. 1a in the form of an inverse pole figure (IPF) map measured using electron backscatter diffraction (EBSD), and from inspection the grain morphology is coarse columnar. This is quantified by the use of image analysis (using the MIPAR™ software package) where the aspect ratios of grains have been determined. For this microstructure, an average aspect ratio of ≈3.1 has been assessed as shown in Fig.1b; it is generally considered that an aspect ratio of magnitude greater than ≈3.0 corresponds to columnar morphologies^15^. Finally, the pole figures obtained from the EBSD experiment from which the degree of texture may be estimated are shown in Fig. 1c. This sample exhibits a moderate texture. For the case of the Ti-12Mo sample, the results of the characterization of its as-deposited microstructure are shown in Fig. 2. From observation of the IPF map (Fig. 2a), the microstructure is coarse columnar, similar to that of the Ti-20V sample, but where the average aspect ratio is ≈4.4 (Fig. 2b), consistent with the assessment made from the IPF map. Finally, from the pole figures (Fig. 2c) deduced from the EBSD characterization, this as-deposited sample exhibits a moderate texture. From these experiments, involving the deposition of powders of the alloys Ti-20V and Ti-12Mo, it is concluded that their grain morphologies are both coarse columnar. These various results and that for the case of Ti-8.5 Cu^5^ are compared in Table 1. Consequently, it appears that the critical value of the growth restriction factor, Q_bin_, above which equiaxed microstructures may be expected lies between 54 K and 62 K. It may also be noted from Table 1 that the Ti-20V and Ti-12Mo samples have relatively low values of ΔT, being ≈10 K and ≈110 K, respectively, whereas the value for the alloy Ti-8.5Cu^5^, is given by ΔT ≈ 603 K, a very significantly larger value. From this assessment, it appears that the critical value of ΔT to realize a CET must be considerably higher than 110 K, and perhaps closer to ≈603 K.

**Table 1:** Grain Morphology, Texture, Q and ΔT values of Ti-20V, Ti-12Mo and Ti-8.5Cu

Alloy	Observed Grain Morphology	Texture<001>β	Growth Restriction Factor, Q(K)	Freezing Range ΔT(K)
Ti-20V	Long, columnar	Strong	0	~10
Ti-12Mo	Long, columnar	Strong	54	~110
Ti-8.5Cu^5^	Equiaxed	Random	62	~603

For the given alloys, the observed grain morphology, the observed textures, and results of estimations of the growth restriction factors (Q), and freezing ranges, (ΔT), as discussed in the text, are noted. The values for the alloy Ti-8.5Cu are taken from the published work on Ti-Cu^5^. The method of calculating Q yields the *Q*_*bin*_ values as noted in the text.

**Fig. 3:**
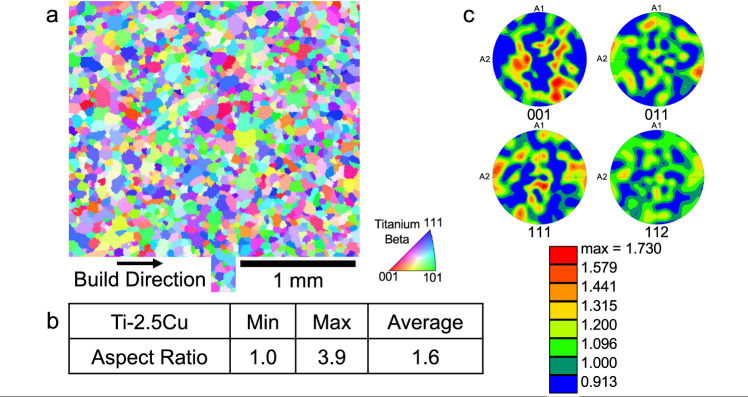
Characterization of the microstructure of the Ti-2.5Cu sample. **a** an IPF map obtained using EBSD to determine the refined equiaxed grain morphology. **b** Results of image analysis to determine the average aspect ratios of the grains, yielding a result consistent with the equiaxed morphology. **c** Pole figures deduced from the EBSD experiment from which a weak texture is observed.

**Fig. 4:**
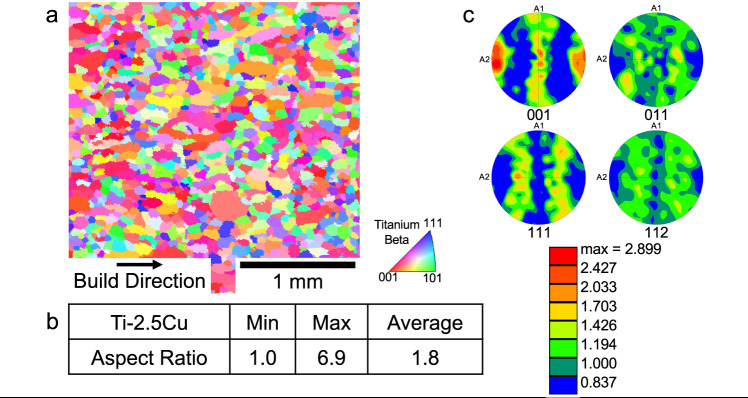
Characterization of the microstructure of the Ti-6.8Cu sample. **a** an IPF map obtained using EBSD to determine the refined equiaxed grain morphology. **b** Results of image analysis to determine the average aspect ratios of the grains, yielding a result consistent with the equiaxed morphology. **c** Pole figures deduced from the EBSD experiment from which a weak texture is observed.

Having assessed that a CET should occur for alloys with values of Q close to, or greater than Q ≈ 62 K, we processed using AM (employing the DED process mentioned above) two Ti-Cu alloys, namely Ti-2.5Cu and Ti-6.8Cu. These were chosen firstly to permit a *direct comparison* with the Ti-8.5Cu alloy^5^, and secondly for their estimated values of Q and ΔT which are listed in Table 2. Thus, values of Q for both these alloys are less than 54 K, such that columnar microstructures would be predicted, whereas the predicted values of ΔT for both of these alloys are very high, namely ≈672 K and ≈623 K, respectively, which leads to a prediction of equiaxed grain morphologies. These two divergent sets of microstructural predictions are included in Table 2. The results of the characterization of the as-deposited powders of these two alloys (Ti-2.5Cu, Ti-6.8Cu) are shown in Fig. 3 and Fig. 4, respectively. For the most dilute alloy, namely Ti-2.5Cu, the IPF map shown in Fig. 3a is consistent with a refined equiaxed grain morphology. The average aspect ratio (Fig. 3b) is 1.6, also consistent with an equiaxed morphology, and the pole figures (Fig. 3c) reveals a somewhat weak texture. For the more concentrated alloy, Ti-6.8Cu, similar results are obtained. Thus, the IPF map (Fig. 4a) is consistent with an equiaxed grain morphology, as is the determination of the average aspect ratio (1.8, Fig. 4b), and the pole figures (Fig. 4c) show a weak texture.

**Table 2:** Observed and predicted grain morphology based on Q and ΔT values of Ti-2.5Cu and Ti-6.8Cu

Alloy	Observed Grain Morphology	Texture < 001 > β	Growth Restriction Factor, Q(K)	Predicted Grain Morphology (based on Q)	Freezing Range ΔT(K)	Predicted Grain Morphology (based on ΔT)
Ti-2.5Cu	Equiaxed	Random	18.2	Long, columnar	~672	Equiaxed
Ti-6.8Cu	Equiaxed	Random	49.6	Long, columnar	~623	Equiaxed

For the two experimental alloys, the observed grain morphology, the observed textures, and results of estimations of the growth restriction factors (Q), and freezing ranges, (ΔT), are given. Also, the predictions of the grain morphologies based on values of Q and ΔT are listed. Again, the method of calculating Q yields the *Q*_*bin*_ values as noted in the text.

**Fig. 5:**
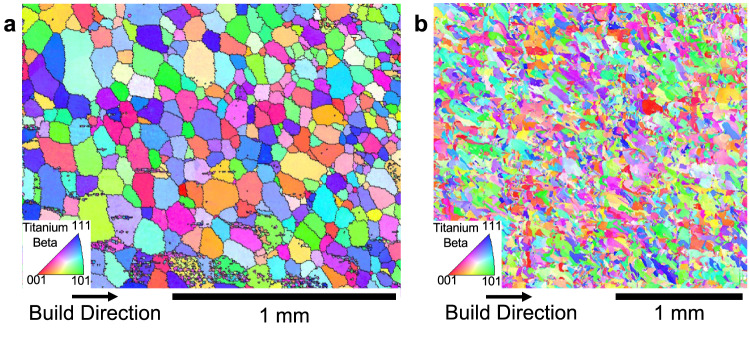
Comparison of microstructures of alloys processed using directed energy deposition (LENS™) and laser powder bed fusion (LPBF). **a** Characterization of the microstructure of the alloy Ti-185 as-built using Directed Energy Deposition (LENS™). **b** Characterization of the microstructure of the alloy Ti-64 + 5Fe as-built using Laser Powder Bed Fusion (LPBF). Note that both alloys exhibit equiaxed microstructures following AM processing.

To examine whether assessments based on the freezing range afforded by alloy compositions are applicable to more complex alloys (i.e., not just the binary alloys considered above), further depositions have been made. These are listed in Table 3, together with their growth restriction factors, freezing ranges, predictions of microstructure based on both growth restriction factors and freezing ranges, and the experimentally observed microstructures. The first three alloys in the Table, i.e., Ti-18 (Ti-5.5Al-5V-5Mo-2.4Cr-0.75Fe-0.15 O), Ti-18 + 3Fe, Ti-185 (Ti-1Al-8V-5Fe) have been processed by LENS™, i.e., the same process as used for the binary alloys described above. The results for the alloys Ti-18 and Ti-18 + 3Fe have been published previously^7^; for the alloy Ti-18, both methods of assessment (growth restriction factors and freezing range) predict columnar microstructures, and the observed microstructure is indeed columnar. However, for the alloy Ti-18 + 3Fe, the prediction of an equiaxed microstructure based on freezing range, is indeed observed. The microstructure of the third alloy, Ti-185, after processing using LENS™ is shown in Fig. 5a, revealing an equiaxed grain morphology, and this is again in agreement with the prediction of microstructure based on freezing range (Table 3); the prediction based on growth restriction factor is incorrect. To assess whether the predictive approach based on freezing range may apply to other methods of additive manufacturing, two alloys, Ti-64 and a modified version Ti-64 + 5Fe, have been processed by Laser Powder Bed Fusion (LPBF). These alloys are also listed in Table 3. The observed microstructure in LPBF builds of Ti-64 consists of large columnar grains, based on numerous reports in the literature^16–20^. From the table, assessments based on both growth restriction factors and freezing range predict this observed microstructure. For the alloy Ti-64 + 5Fe, following processing using LPBF, the observed microstructure is shown in Fig. 5b and appears to be equiaxed. This has been confirmed using image analysis, where a grain aspect ratio of 2.4 has been determined which is well below the threshold of 3.0 for equiaxed microstructures^15^. As shown in Table 3, the prediction of microstructure based on freezing range is correct whereas that based on growth restriction factors is incorrect. Regarding AM of Ti alloys, it appears that predictions of microstructure based on freezing range applies at least to both blown powder and powder bed techniques.

**Table 3:** Observed and predicted grain morphology based on Q and ΔT values for five different multi-component Ti alloys

Alloy	Observed Grain Morphology	Growth Restriction Factor, Q(K)	Predicted Grain Morphology (based on Q)	Freezing Range ΔT(K)	Predicted Grain Morphology (based on ΔT)
Ti-18	Columnar	29.0	Columnar	~125	Columnar
Ti-18 + 3Fe	Equiaxed	40.4	Columnar	~519	Equiaxed
Ti-185	Equiaxed	19.0	Columnar	~490	Equiaxed
Ti-64 (LPBF)	Columnar	8.0	Columnar	~153	Columnar
Ti-64 + 5Fe (LPBF)	Equiaxed	27.0	Columnar	~515	Equiaxed

Application of the two methods (i.e., Growth Restriction Factor or Freezing Range) for predicting the grain morphology of the as-deposited practical alloys listed. The first three alloys were deposited using the LENS™ (directed energy deposition, blown powder) and last two alloys were deposited using LPBF (laser powder bed fusion). For the five alloys, the observed grain morphologies, and the results of estimations of the growth restriction factors (Q), and freezing ranges, (ΔT), are given. Also, the predictions of the grain morphologies based on values of Q and ΔT are listed. Again, the method of calculating Q yields the *Q*_*bin*_ values as noted in the text.

In this work, we have shown from the comparisons of the predictions of as-deposited grain morphologies given in Tables 2 and 3 for the alloys considered, it appears that assessments based on the freezing range afforded by the alloy compositions, rather than on the growth restriction factors, provide a more accurate approach to such microstructural predictions. As noted above, the notion of the importance of freezing range arises from a consideration of the influence of the relatively rapid rate of heat extraction imposed by AM techniques such as LENS™ and powder bed fusion. Thus, a relatively large degree of undercooling prior to solid nucleation may be achieved if the freezing range is significant. The notion of the importance of growth restriction factors is based on the detailed work involving grain refinement in castings^13^. The difference in the imposed cooling rates in these two processing routes, i.e., castings vs. AM techniques, is very significant, and may well be the basis for the divergence in predictive capabilities. Of course, the application of both approaches, freezing range and growth restriction factors, depends on the accuracy of the predicted phase diagrams by the various computational thermodynamics techniques, and therefore on the quality of the databases used in these computational techniques.

## Methods

### Laser directed energy deposition

Elemental powders of Ti, V, Mo and Cu were procured from Alfa Aesar, USA. The nominal compositions (in wt. %) of the four β-Ti alloys that were additively manufactured are Ti-20V, Ti-12Mo, Ti-2.5Cu and Ti-6.8Cu. An Optomec LENS-750 system equipped with an IPG YLS-1500 fiber laser system (maximum power output of 1500 W) was used to deposit composite blocks with dimensions of 25.4 × 25.4 × 25.4 mm. The depositions were carried out with a single powder hopper loaded with the roller-mixed feedstock compositions. The LENS-750 processing parameters were: 600 W laser power; 0.5 mm diameter laser beam on the sample surface; 12.7 mm/s laser scan speed; 0.254 mm vertical layer spacing; 0.381 mm hatch width with 90° rotation in the hatch direction between layers. This combination of laser parameters provided an input energy fluence of 94.48 J/mm^2^. The input energy fluence was calculated using the expression *E* = *P* */* *V* *×* *D*, where P is the laser power, V is the scan speed, and D is the laser beam diameter. The oxygen level in the glove box was maintained below 10 ppm.

### Laser powder bed fusion

Ti-6Al-4V powder procured from Carpenter, was premixed with 5 wt.% Fe in a roller for 12 h. A Trumpf TruPrint 1000 was used for the fabrication of a 20 mm*20 mm*10 mm part. The parameters used were 180 W power, 600 mm/s travel speed, 30-micron layer thickness, and 60-micron hatch spacing. Bidirectional stripes (90^o^ rotation after each layer) scan strategy was employed.

### Microstructure characterization

The deposited builds were separated from the Ti64-seed plate. A thin section (parallel to the build direction) was sliced from each deposit using a KENT USA (WSI-200) electric discharge machine (EDM) and subsequently polished using traditional polishing procedures for scanning electron microscopy analysis. EBSD was performed on a Thermo Fisher Scientific Apreo SEM equipped with an EDAX Hikari EBSD camera. The accelerating voltage was 20 kV and the beam current was 26 nA. Large area scans were performed where a number of scans have been stitched and merged together. The grains were reconstructed from the EBSD data using the software developed by Pilchak^21^. The texture of the grains was determined using EDAX OIM Analysis software. The MIPAR™ software package was used to determine the grain aspect ratio.

## Supplementary information

Peer Review File

## Data Availability

The data generated in this study are available from the corresponding authors upon request.
